# Integrated mechanisms of CaMKII-dependent ventricular remodeling

**DOI:** 10.3389/fphar.2014.00036

**Published:** 2014-03-12

**Authors:** Michael M. Kreusser, Johannes Backs

**Affiliations:** ^1^Research Unit Cardiac Epigenetics, Department of Cardiology, University of HeidelbergHeidelberg, Germany; ^2^German Center for Cardiovascular Research (DZHK)Partner Site Heidelberg/Mannheim, Germany

**Keywords:** CaMKII, epigenetics, transcription factors, HDAC4, remodeling

## Abstract

CaMKII has been shown to be activated during different cardiac pathological processes, and CaMKII-dependent mechanisms contribute to pathological cardiac remodeling, cardiac arrhythmias, and contractile dysfunction during heart failure. Activation of CaMKII during cardiac stress results in a broad number of biological effects such as, on the one hand, acute effects due to phosphorylation of distinct cellular proteins as ion channels and calcium handling proteins and, on the other hand, integrative mechanisms by changing gene expression. This review focuses on transcriptional and epigenetic effects of CaMKII activation during chronic cardiac remodeling. Multiple mechanisms have been described how CaMKII mediates changes in cardiac gene expression. CaMKII has been shown to directly phosphorylate components of the cardiac gene regulation machinery. CaMKII phosphorylates several transcription factors such as CREB that induces the activation of specific gene programs. CaMKII activates transcriptional regulators also indirectly by phosphorylating histone deacetylases, especially HDAC4, which in turn inhibits transcription factors that drive cardiac hypertrophy, fibrosis, and dysfunction. Recent studies demonstrate that CaMKII also phosphorylate directly histones, which may contribute to changes in gene expression. These findings of CaMKII-dependent gene regulation during cardiac remodeling processes suggest novel strategies for CaMKII-dependent “transcriptional or epigenetic therapies” to control cardiac gene expression and function. Manipulation of CaMKII-dependent signaling pathways in the settings of pathological cardiac growth, remodeling, and heart failure represents an auspicious therapeutic approach.

## Introduction

Heart failure is the leading cause of death in developed countries and is characterized by adverse cardiac remodeling upon pathological stress situations such as arterial hypertension, ischemic injuries or due to genetic causes. Adverse left ventricular remodeling is usually described by a combination of myocardial hypertrophy, cell death, interstitial fibrosis and an activation of a so-called fetal gene program (Koitabashi and Kass, 2012). Calcium-dependent signaling pathways including Calcium/Calmodulin-dependent kinase II (CaMKII) signaling play pivotal roles in adverse cardiac remodeling (Heineke and Molkentin, 2006; Bers, 2008; Backs et al., 2009; Ling et al., 2009). Activation of CaMKII during cardiac stress results in a broad number of biological effects. On the one hand, CaMKII mediates immediate effects due to phosphorylation of cellular proteins such as ion channels and calcium handling proteins (Anderson et al., 2011). On the other hand, CaMKII affects structural features of the cardiac phenotype due to phosphorylation of proteins of the transcriptional machinery (Anderson et al., 2011). This review focuses on CaMKII-dependent transcriptional and epigenetic mechanisms that occur in cardiomyocytes during pathological and physiological processes. First, we will review the different CaMKII genes and splice variants that localize to different subcellular compartments.

## CaMKII isoforms and splice variants

In 2003, Colomer and colleagues observed an increased activity of Calcium/Calmodulin-dependent kinases upon pathological pressure overload due to transverse aortic constriction (TAC), and they described the expression patterns of the Calcium/Calmodulin-dependent kinases CaMKI, CaMKII, and CaMKIV (Colomer et al., 2003). They found CaMKI to be expressed in left ventricular tissue, but not up-regulated upon TAC. Whereas artificial overexpression of CaMKIV in a transgenic model was sufficient to induce cardiac hypertrophy in another study (Passier et al., 2000), in the model of Colomer, left ventricular CaMKIV was not detectable, and mice lacking CaMKIV did not display an altered response to TAC, indicating that CaMKIV is not required for cardiac hypertrophy. They convincingly demonstrated that CaMKII is the only multifunctional CaMK that is not only up-regulated on the expression level but also activated after TAC.

CaMKII consists of four different isoforms with distinct expression patterns. CaMKIIα and CaMKIIβ are enriched in neuronal tissue, and CaMKIIδ, and CaMKIIγ are expressed ubiquitously (Hudmon and Schulman, 2002). CaMKIIδ is the most abundant cardiac CaMKII isoform but CaMKIIγ is also expressed in the heart (Hoch et al., 1999; Colomer et al., 2003). The first *in vivo* studies establishing CaMKII as a potential target for cardiac arrhythmias and structural heart disease were conducted by the use of a pharmacological inhibitor such as KN-62 or KN-93 and a CaMKII inhibitory peptide (Zhang et al., 2005; Vila-Petroff et al., 2007; Liu et al., 2011). Due to the unclear role of the single CaMKII isoforms and potential unspecific effects of CaMKII inhibitors, isoform-specific genetic loss of function models were generated. Mice with a global deletion of CaMKIIδ were protected against adverse cardiac remodeling (Backs et al., 2009; Ling et al., 2009). CaMKIIδ global knockout mice produced by us were protected from cardiac fibrosis and hypertrophy 3 weeks after TAC surgery. CaMKIIδ global knockout model generated by Ling and colleagues were protected from fibrosis and dysfunction. These mice were not protected from cardiac hypertrophy 2 weeks but only 6 weeks after TAC. These seemingly different phenotypes with regard to cardiac hypertrophy may be explained by different surgery techniques, different genetic backgrounds, or different knockout strategies. With regard to the latter, in the first model, no residual protein was expressed (transcriptional null due to deletion of exon 1 and 2), whereas in the second model the possible existence of a truncated protein encoding a region before exon 8 was not ruled out (exons 9–11 were deleted). The specific role of cardiac CaMKIIγ and a potential redundancy with CaMKIIδ have not been investigated yet. In human and experimental heart failure, enhanced CaMKII activity was mainly attributed to an enhanced expression of the CaMKIIδ splice variants CaMKIIδB and CaMKIIIδC (Edman and Schulman, 1994; Hoch et al., 1999). From transgenic mouse models with artificial overexpression of these splice variants it was concluded that CaMKIIδB (localizes to the nucleus) promotes cardiac hypertrophy and CaMKIIδC (localizes to the cytosol) results in dilated cardiomyopathy, respectively (Zhang et al., 2002b, 2003). Moreover, CaMKIIδ A (localizes to sarcolemmal and nuclear membranes) was implied as another splice variant that is regulated at least in a model of cardiac hypertrophy due to isoproterenol treatment in mice (Xu et al., 2005; Li et al., 2011). However, to our knowledge transgenic models of CaMKIIδ A have not been generated so far. An overview of available genetic mouse models related to cardiac CaMKII is given in Table 1.

**Table 1:** **Genetic mouse models for CaMKIIδ and γ**.

**Gene/splicing variant**	**Type**	**Strategy**	**Cardiac phenotype**	**Location**	**References**
CaMKIIδ*B*	Gain of function	α*MHC*-driven transgene	Cardiac hypertrophy	Nucleus	Zhang et al., 2002b
CaMKIIδ*C*	Gain of function	α*MHC*-driven transgene	Dilated cardiomyopathy	Cytosol	Zhang et al., 2003
CaMKIIδ	Loss of function	Global knockout exons 9–11	Protection from fibrosis, dysfunction, and late hypertrophy	Nucleus/Cytosol	Ling et al., 2009
CaMKIIδ	Loss of function	Global knockout exons 1–2	Protection from early hypertrophy and fibrosis	Nucleus/Cytosol	Backs et al., 2009
CaMKIIγ	Loss of function	Global knockout exons 1–2	Not investigated	Nucleus/Cytosol	Backs et al., 2010

Cardiomyocyte-specific transgenic overexpression of CaMKIIδ (splice variants B and C) are driven by the αMHC promoter. Global knockout models for CaMKIIδ were generated by two labs. The second cardiac CaMKII isoform, CaMKIIγ, has so far not been investigated with regard to cardiac stress situations.

## CaMKII and transcriptional regulation

**Figure 1:**
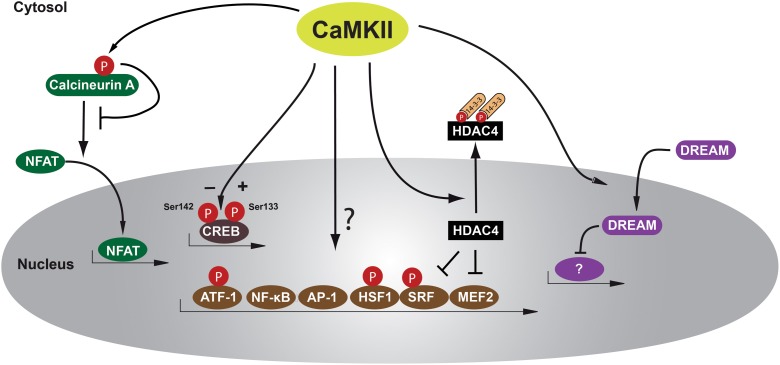
**Schematic of transcription factors and transcriptional repressors regulated by CaMKII in cardiomyocytes**. CaMKII phosphorylates HDAC4 at Ser-467 and Ser-632, allowing binding of the chaperone protein 14-3-3, leading to nucleo-cytoplasmic shuttling of a phospho-HDAC4/14-3-3 complex out of the nucleus and resulting in derepression of transcription factors such as MEF2 that regulates genes responsible for adverse cardiac remodeling. Other transcription factors such as NF-κB or HSP-1 may play maladaptive or adaptive roles and these factors can be directly or indirectly regulated by CaMKII. Another transcription factor regulated after β-adrenergic stimulation is CREB that can be phosphorylated by CaMKII at two serine residues, Ser-133 and Ser-142, resulting in opposing effects in regard to activation of CREB. However, the functional effects of CREB/CaMKII interaction during cardiac remodeling remain unclear. Another recently recognized mechanism is translocation of the transcriptional repressor DREAM from the cytosol to the nucleus. Calcineurin/NFAT interaction may also be inhibited by direct phosphorylation by CaMKII at Ser-411, leading to decreased NFAT translocation to the nucleus and subsequent reduced transcriptional activity. AP-1 activation protein-1, ATF-1 activating transcription factor-1, CaMKII Calcium/Calmodulin-dependent kinase II, CREB cAMP-response element binding protein, DREAM downstream regulatory element agonist modulator, HDAC4 histone deacetylase 4, HSF-1 heat shock factor 1, MEF2 myocyte elongation factor, NFAT nuclear factor of activated T-cells, NF-κB nuclear factor κB, and SRF serum response factor.

Effects of CaMKII on cardiac gene expression was first reported by the group of Joan Heller Brown when transient expression of CaMKIIδB in neonatal rat ventricular myocytes induced gene expression of atrial natriuretic factor (ANF) and resulted in enhanced transcriptional activation of an ANF-luciferase reporter gene (Ramirez et al., 1997). As we know now, CaMKII is involved in the regulation of many transcription factors such as the activation protein-1 (AP-1) (Antoine et al., 1996), activating transcription factor-1 (ATF-1) (Shimomura et al., 1996), serum response factor (SRF) (Fluck et al., 2000), cAMP-response element binding protein (CREB) (Sun et al., 1994), and myocyte enhancer factor 2 (MEF2). The latter is discussed in the next paragraph. An overview about the identified transcriptional regulators is given in Figure 1.

A well-known transcription factor in striated muscle biology is SRF, and Calcium/Calmodulin-dependent regulation of SRF via interaction with CaMKIV and histone deacetylase 4 (HDAC4), but not CaMKII, was demonstrated to be involved in the development of cardiac hypertrophy (Davis et al., 2003). A direct phosphorylation of SRF by CaMKII has been shown in skeletal muscle at Ser-103 and Thr-160 (Fluck et al., 2000), but its relevance in cardiomyocytes remains unclear. However, data from other cell types indicate that SRF-dependent gene transcription might depend on phosphorylation by CaMKII (Ely et al., 2011). As genetic animal models provide evidence for an important role for SRF in the induction and maintenance of the cardiac myogenic program (Lin et al., 1997; Parlakian et al., 2005; Backs et al., 2011), a better understanding of CaMKII-dependent SRF regulation is urgently needed.

AP-1 elements are a group of transcription factors composed of either homodimers of the Jun family (c-Jun, JunB, and JunD) or heterodimers of the Fos and Jun families (c-Fos, Fra-1, Fra-2, and FosB) (Mechta-Grigoriou et al., 2001). There is evidence for CaMKII-dependent transcriptional activity via AP-1 in non-cardiac tissues (Mishra et al., 2005; Zayzafoon et al., 2005). Recently, CaMKII-dependent upregulation of the sodium/calcium exchanger 1 (NCX1) has been shown to depend on AP-1 transcription factors c-Jun and JunB in cardiomyocytes (Mani et al., 2010). However, it remains unclear whether CaMKII phosphorylates AP-1 elements directly or indirectly.

As one of the well characterized transcription factors activated by G-protein coupled receptors, CREB has been shown to be phosphorylated by Calcium/Calmodulin-dependent kinases (Sheng et al., 1991). Several phosphorylation sites in CREB have been identified to regulate transcriptional activity and have been shown to be regulated by CaMKII in a dual way. Phosphorylation of CREB at Ser-133 by PKA and CaMKII is required for CREB activation, whereas phosphorylation of Ser-142 by CaMKII inhibits CREB activity by inhibition of CREB dimerization and protein/protein interactions that are necessary to form an active promoter complex (Wu and McMurray, 2001). *In vitro* studies using purified CaMKIV and CaMKII have demonstrated that CaMKIV phosphorylates CREB on Ser-133, whereas CaMKII has equal affinity for Ser-133 and Ser-142 (Sun et al., 1994). However, whereas nuclear calcium elevations increase CREB-dependent transcriptional activity (Kobrinsky et al., 2011), it remains unclear whether CaMKII-dependent CREB phosphorylation plays a significant role in cardiac remodeling processes (Li et al., 2006). Another member of the cAMP-responsive transcription factor family is ATF-1. ATF-1 is phosphorylated at Ser-63 by CaMKII, increasing its transcriptional activity (Shimomura et al., 1996), but the relevance of ATF-1 activity in cardiomyocytes is unknown.

Studies using KN-93, a CaMKII-inhibitory chemical compound, postulated CaMKII-dependent activation of transcription factor nuclear factor-κB (NF-κB) leading to cardiomyocyte hypertrophy (Kashiwase et al., 2005). In CaMKIIδ knockout mouse studies, NF-κB-dependent mechanisms and subsequent activation of inflammatory genes were found to play a maladaptive role in myocardial ischemia and ischemia/reperfusion injury in mice (Singh et al., 2009; Ling et al., 2013). Of note, the latter work conducted by the group of Mark Anderson was the first to perform gene expression profiling in a relevant animal model of cardiac stress, after myocardial infarction in mice. In this study, a CaMKII inhibitory peptide was used. Thus, potential off target effects need to be taken into account. For instance, it was shown that the CaMKII inhibitory peptide can also inhibit protein kinase D (PKD) (Backs et al., 2009). Gene expression arrays in genetic loss of function models might provide important additional information.

A possible physiological CaMKII-dependent transcriptional effect mediated by CaMKIIδB is the activation of heat shock factor 1 (HSF-1), a transcription factor responsible for inducible heat shock protein 70 (iHSP70) gene regulation. CaMKII is known to phosphorylate HSF-1 at Ser-230 (Holmberg et al., 2001), and Wei Peng and colleagues provided data suggesting that this might be a CaMKII-dependent antiapoptotic mechanism during cardiac ischemia and reperfusion (Peng et al., 2010). In this context, others could show that CaMKIIδB protects from doxorubicine-induced apoptosis, perhaps through GATA4-related expression of the antiapoptotic bcl-2 (B-cell lymphoma 2) gene (Little et al., 2009), although the mechanism how CaMKII induced GATA4-dependent gene expression was not shown. Taken the central role of GATA4 in cardiac hypertrophy and growth (Oka et al., 2006; Heineke et al., 2007), more data are warranted to clarify the role of GATA4 as a downstream target of CaMKII. Taken together, there are only sparse data available on how CaMKII directly interacts with transcription factors, and for most factors a direct binding and phosphorylation by CaMKII is not yet shown (See also Table 2).

**Table 2:** **CaMKII-dependent regulators of cardiac transcription**.

**Name**	**Abbrev.**	**Type**	**Effect**	**Phosphorylation site**	**Kinase assay**	**References**
cAMP-response element binding protein	CREB	Transcription factor	Unknown	Ser-133, Ser-142	Yes	Sun et al., 1994
Activating transcription factor 1	ATF-1	Transcription factor	Unknown	Ser-63	Yes	Shimomura et al., 1996
Myocyte elongation factor 2	MEF2	Transcription factor	Hypertrophy/remodeling	Unknown	/	Passier et al., 2000
Serum response factor	SRF	Transcription factor	Unknown	Ser-103, Thr-160	Yes	Fluck et al., 2000
Nuclear factor κB	NF-κB	Transcription factor	Hypertrophy/remodeling	Indirect via IκB kinase	/	Kashiwase et al., 2005; Ling et al., 2013
Histone deacetylase 4	HDAC4	Transcriptional repressor	Hypertrophy/remodeling	Ser-467, Ser-632	Yes	Backs et al., 2006
Histone deacetylase 5	HDAC5	Transcriptional repressor	Hypertrophy/remodeling	Unknown	/	Wu et al., 2006; Backs et al., 2008
GATA4	/	Transcription factor	Antiapoptotic	Unknown	/	Little et al., 2009
Activation protein 1	AP-1	Transcription factor	Calcium homeostasis	Unknown	/	Mani et al., 2010
Heat shock factor 1	HSF-1	Transcription factor	Antiapoptotic	Ser-230	Yes	Holmberg et al., 2001; Peng et al., 2010
Downstream regulatory element agonist modulator	DREAM	Transcriptional repressor	Calcium homeostasis	Unknown	/	Ronkainen et al., 2011
Histone H3	H3	Histone	Hypertrophy/remodeling	Ser-10	Yes	Awad et al., 2013

CaMKII interacts with various transcription factors, transcriptional repressors, and histone 3 and thereby influences cardiac gene expression. This interaction can be a direct phosphorylation of Ser/Thr residues by CaMKII, indirect via other proteins (other kinases or cardiac repressors) or by unknown mechanisms. Known phosphorylation site and proof of direct phosphorylation are indicated. CaMKII Calcium/Calmodulin-dependent kinase II.

In an interesting recent *in vitro* study, Jarkko Ronkainen et al. describe how CaMKII potentiates the translocation of the transcriptional repressor DREAM (downstream regulatory element agonist modulator) into the nucleus and thereby promotes DREAM-induced transcriptional repression. In their study, the authors could show that this mechanism is involved in CaMKII-dependent downregulation of the pore-forming α-subunit (Cav1.2) of the *L*-type calcium channel (LTCC) and postulate this to be a physiological feedback mechanism, which enables cardiomyocytes to adjust calcium influx through the LTCC to calcium-activated CaMKII activity (Ronkainen et al., 2011).

Another “indirect” transcriptional mechanism seems to be mediated by an interaction between CaMKII and calcineurin A. The phosphatase calcineurin A dephosphorylates nuclear factor of activated T-cells (NFAT), resulting in nuclear accumulation of NFAT and consequent activation of NFAT-dependent transcriptional programs and severe cardiac hypertrophy (Molkentin et al., 1998). In an elegant study, it was demonstrated that cytosolic CaMKIIδC phosphorylates calcineurin A within its calmodulin binding domain at Ser-411 and thereby inhibits its activity (MacDonnell et al., 2009). Although the relevance of these findings needs to be proven *in vivo*, this suggest that CaMKIIδC may act as a negative modulator of calcineurin/NFAT activity.

## CaMKII and epigenetic regulation

Besides its direct effects on transcriptional regulators, CaMKII regulates gene expression also by phosphorylation of proteins of the epigenetic machinery, especially histone deacetylases (HDACs) and in particular class II HDACs. These interesting mechanisms were initially identified upon the observation that class II HDACs interact with the transcription factor MEF2. MEF2 was introduced to depend on CaMKI and CaMKIV more than 10 years ago by the group of Eric Olson and has been established as a critical transcription factor in cardiac remodeling processes (Passier et al., 2000). MEF2 is a common target for several hypertrophic pathways, although its precise function in cardiac remodeling and the cardiac genes that are modulated by this factor are still under investigation. MEF2 proteins are responsive to calcium-controlled signaling pathways, such as CaMKI, CaMKII, CaMKIV, and Calcineurin (Passier et al., 2000; McKinsey et al., 2002; Zhang et al., 2007). Class II HDACs are expressed in the heart and contain a MEF2 binding domain in the N-terminal region, which is not present in other HDACs. This N-terminal domain binds to the chaperone 14-3-3 and is then exported from the nucleus with the consequent de-repression of MEF2 (Backs and Olson, 2006; McKinsey, 2007; Ling et al., 2013). 14-3-3 binding depends on phosphorylation of HDACs by different kinases. For example, PKD phosphorylates all class II HDAC family members (HDAC4, HDAC5, HDAC7, HDAC9) (Vega et al., 2004; Harrison et al., 2006).

**Figure 2:**
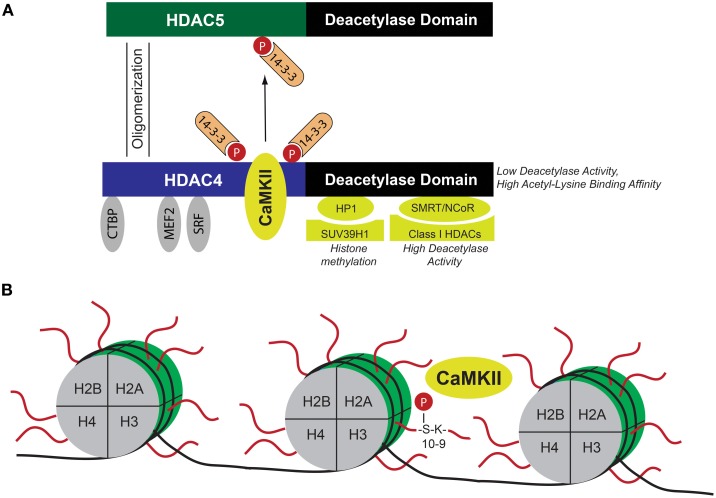
**Schematic of CaMKII-dependent epigenetic mechanisms. (A)** HDAC4 as a nodal point for CaMKII-dependent epigenetic regulation. CaMKII binds to HDAC4 and phosphorylates HDAC4 at Ser-467 and Ser-632, leading to nucleo-cytoplasmic shuttling of HDAC4. When located in the nucleus, HDAC4 represses transcription factors such as MEF2, SRF, or the co-repressor CtBP. Binding to these transcriptional regulators directs HDAC4 to specific chromatin regions. HDAC4 recruits other chromatin modifying enzymes and direct them to the aforementioned specific chromatin regions. This results in CaMKII/HDAC4-dependent regulation of histone methylation (via interaction with HP1 and histone methyltransferase SUV39H1) and deacetylation (via interaction with SMRT/N-CoR and class I HDACs). Moreover, HDAC4 oligomerizes with HDAC5, and thereby induces 14-3-3 dependent nucleo-cytoplasmic shuttling of the HDAC4/HDAC5 complex, leading to de-repression of HDAC4/HDAC5-dependent transcription factors. Thus HDAC4 integrates CaMKII-dependent signals via epigenetic mechanisms. **(B)** Histones (H2A, H2B, H3, and H4) assemble with DNA to form nucleosomes. CaMKII directly phosphorylates Ser-10 in the N-terminal region of histone 3, which is located next to Lys-9, a major site for acetylation, and methylation. Phosphorylation at Ser-10 was suggested to result in cardiomyocyte hypertrophy and increased chromatin binding of CaMKII at specific gene loci reactivated during cardiac hypertrophy. CaMKII Calcium/Calmodulin-dependent kinase II, CtBP C-terminal binding protein, HDAC histone deacetylase, HP1 heterochromatin protein 1, MEF2 myocyte elongation factor, N-CoR nuclear receptor co-repressor, SMRT silencing mediator of retinoic acid and thyroid hormone receptor, and SRF serum response factor.

We found that CaMKII selectively signals to HDAC4 via binding to a unique docking site and phosphorylation of Ser-467 and Ser-632 (Backs et al., 2006) (See also Figure 2). These data were confirmed by others and phosphorylation of HDAC4 by CaMKII was suggested as a central mechanism in the development of cardiac hypertrophy and remodeling (Little et al., 2007; Zhang et al., 2007; Backs et al., 2009). HDAC5 does not bind to CaMKII and can therefore only be regulated by CaMKII when it is located in close proximity to HDAC4. When HDAC5 oligomerizes with HDAC4, it can be phosphorylated and exported in a complex with HDAC4 and CaMKII (Backs et al., 2008). Accordingly, HDAC5 has been shown to be regulated by CaMKII under certain conditions. The Bers lab demonstrated that calcium in the nuclear envelope is regulated independently from the global calcium transients that cause contraction at each heartbeat. Interestingly, calcium release from the nuclear envelope activates nuclear CaMKII, which triggers nuclear export of HDAC5 (Wu and Bers, 2006; Wu et al., 2006). Whereas nuclear CaMKIIδB and cytosolic CaMKIIδC exert different effects on the phosphorylation of calcium handling proteins as the ryanodine receptor or phospholamban and on calcium homeostasis (Zhang et al., 2007), both isoforms lead to cytosolic accumulation of HDAC4 and an increase in the activity of the transcription factor MEF2 (Backs et al., 2006). Nuclear CaMKIIδB phosphorylates HDAC4 in the nucleus, leading to nucleo-cytoplasmic shuttling of HDAC4. Activation of cytoplasmic CaMKIIδC phosphorylates HDAC4 in the cytosol and prevents the import of HDAC4 from the cytosol to the nucleus (Backs et al., 2006). Thus, cytoplasmic CaMKII is also capable to regulate transcription in addition to its effects on excitation-contraction coupling. These findings strongly suggested that CaMKII indirectly regulates MEF2 by dissociating HDAC4 and HDAC5. However, HDAC4 binds to many other proteins such as other transcription factors including SRF (Davis et al., 2003), co-repressors as CtBP (C-terminal-binding protein) (Zhang et al., 2001) but also to other chromatin modifying enzymes (Zhang et al., 2002a), opening the possibility that CaMKII exerts via cytosolic accumulation of HDAC4 broader effects than simply activating MEF2 (Lehmann et al., 2013).

Histones are major components of chromatin and assemble with DNA to form nucleosomes, (Jenuwein and Allis, 2001). The N-terminal regions of histones are subjected to a variety of post-translational modifications, including acetylation, methylation, ubiquitination, SUMOylation, and phosphorylation (Kouzarides, 2007; Ruthenburg et al., 2007). Figure 2 summarizes important interacting proteins of HDAC4 that direct HDAC4 to specific chromatin regions by binding to transcription factors. Of note, the deacetylase activity of class II HDACs is low but the binding affinity to acetylated lysines is high, suggesting that HDAC4 is mainly recruited to acetylated chromatin regions in close proximity to regions where MEF2 or SRF binds (Lahm et al., 2007). Although its deacetylase activity is low, HDAC4 binds indirectly via the co-repressors SMRT (silencing mediator of retinoic acid and thyroid hormone receptor) and N-CoR (nuclear receptor co-repressor) to class I HDACs with high deacetylase activity (Fischle et al., 2002) and via HP1 (heterochromatin protein 1) to histone methyltransferases (Zhang et al., 2002a). Thus it is tempting to speculate that CaMKII effects besides transcriptional activity of MEF2 and SRF also class I HDAC-dependent histone acetylation and histone methyltransferase-dependent histone methylation. Indeed, together with the Maack lab we could show that HDAC4 controls histone methylation in a CaMKII-dependent manner (Hohl et al., 2013). ANF and brain natriuretic peptide (BNP) expression in failing hearts was accompanied by demethylation of histone 3 at lysine 9 (H3K9) and dissociation of HP1 from the promoter regions of ANF and BNP, and this was controlled by HDAC4, possibly by forming a transcriptional repressor complex with the histone methyltransferase SUV39H1 that was disrupted by CaMKII-induced phosphorylation of HDAC4. The importance of the CaMKII/HDAC4/MEF2-pathway with regard to epigenetic mechanisms in cardiac remodeling was underscored by a recent study from the Condorelli lab (Papait et al., 2013). The authors performed chromatin immunoprecipitation combined with genomic sequencing (ChIP-Seq) and RNA sequencing in isolated cardiomyocytes after TAC surgery and found a specific epigenetic signature that regulated gene expression by governing the activity of promoters and enhancers related to cardiac hypertrophy. Interestingly, they found MEF2 to be the main transcription factor to orchestrate this hypertrophic gene program by regulating the activity of transcriptional enhancers.

With regard to epigenetic mechanisms, in cardiac biology most attention so far was paid to histone acetylation and methylation. Histone phosphorylation is thought to be important for cell cycle regulation and was thus not carefully studied in the adult heart (Walter et al., 2008; Baek, 2011). Recently, it was reported by the group of Coralie Poizat that nuclear CaMKII activates cardiac transcription by direct binding to the chromatin. CaMKII was shown to phosphorylate Ser-10 of histone 3 (H3S10) which is located next to Lys-9, a major site for acetylation and methylation (Awad et al., 2013). Phosphorylation of H3S10 was accompanied by hypertrophy of primary cultured cardiomcyocytes and with increased chromatin binding of CaMKII at specific gene loci reactivated during cardiac hypertrophy under control of the transcription factor MEF2. These findings represent an interesting new epigenetic mechanism governed by CaMKII. The possibility, that ventricular remodeling can be mediated by CaMKII-dependent chromatin modifications opens a new avenue of regulatory mechanisms. Important further studies are warranted. ChIP-Seq studies may identify direct target genes of CaMKII that are important for diseases processes.

## Summary and outlook

CaMKII regulates not only immediate cellular functions but also chronic processes such as ventricular remodeling leading to heart failure. In particular, CaMKII integrates several cellular pathways by inducing gene programs that are not understood in detail. Here, we reviewed the yet known transcriptional and epigenetic mechanisms by which CaMKII regulates cardiac gene expression. However, the relative importance of the different downstream mechanisms still needs to be clarified. Unbiased gene expression analyses and epigenetic profiling are warranted to define the specific gene programs that contribute to phenotypic changes induced by CaMKII.

### Conflict of interest statement

The authors declare that the research was conducted in the absence of any commercial or financial relationships that could be construed as a potential conflict of interest.
